# ‘There is no more future for me? Like really, are you kidding?’: agency and decision-making in early motherhood in an urban area in Johannesburg, South Africa

**DOI:** 10.1080/16549716.2021.1886456

**Published:** 2021-03-03

**Authors:** Nirvana Pillay

**Affiliations:** School of Public Health, Faculty of Health Sciences, University of the Witwatersrand, Johannesburg, South Africa

**Keywords:** Teenage pregnancy, agency, health, education, social support, kinship

## Abstract

**Background**: The South African development goals for young women aged 15 to 24 are to reduce HIV incidence, teenage pregnancy and gender-based violence, and to increase school completion and economic security. Early, unintended pregnancy undermines these goals, creating discourses of early motherhood that position young women as powerless. There has been scant attention on the agency of young women in their structural context.

**Objective**: This study explored how young women exercise agency after an unintended pregnancy and make decisions concerning their future, including sexual and reproductive health, school completion and/or income generation, and caregiving for their babies.

**Methods**: I used narrative analysis to explore the lived experiences of young mothers, paying attention to decision-making during pregnancy and motherhood. Domains of analysis included health care, education, and caregiving. I conducted in-depth interviews with 30 young mothers: 30 were interviewed once, nine were interviewed twice, and six were interviewed three times. I interviewed four significant people in the lives of young mothers and six health care providers at a health centre.

**Results**: Progressive policy facilitates increased access to services for young pregnant and parenting women. However, education and health care providers continue to discriminate against them, formally through denying them access to services and informally through discourses of shame which pervade their structural context. Kinship capital in urban and rural contexts and the Child Support Grant mitigate some struggles in early motherhood and help young mothers navigate decision-making.

**Conclusion**: Young mothers exercise agency along a continuum to realise their aspirations. Social and structural support mediate their agency. Policy needs to expand the focus from prevention to include issues of care and support after an early, unintended pregnancy to ensure the health and wellbeing of young mothers and their children.

## Background

The South African development agenda for young pregnant and parenting women is progressive. Policy and legislative frameworks protect their right to health, education, and social protection. These policies are aligned with the Constitution of South Africa [1] and the National Development Plan [2], which reflects the Sustainable Development Goals [3] in various respects. The National Strategic Plan for HIV, AIDS and TB (NSP 2017–2022) identifies adolescent girls and young women (15–24 years) as vulnerable, and aims to decrease new HIV infections, teenage pregnancy, and sexual and gender based violence, and increase retention in school and economic empowerment [4]. The prevention of unintended pregnancy is therefore part of a multi-layered, multi-stakeholder development agenda.

Early motherhood is defined by the World Health Organization as childbearing before age 20 [5]. The public health focus on early, unintended pregnancy is premised on correlations between lower age and increased risk of adverse health consequences for young mothers and their babies, particularly so for mothers under 15 [5]. Globally, 16 million women between 15 and 19 years give birth annually (11% of all births) [6]. In South Africa, in spite of decreasing fertility levels, there has only been a slight decrease in rates of pregnancy for women 15–19 years who had ever given birth from 13.2% in 1998 to 12.4% in 2016, and 16.4% to 15.6% of those who had ever been pregnant [7,8]. It is estimated that 27.8% of women under 19 have begun childbearing [8,p.12].

Social and economic disadvantage is recognised as a cause and consequence of early motherhood [9–13]. Public health evidence has stimulated broader social discourse on the disadvantages to young mothers and their babies by early, mistimed reproduction. This discourse is created when scientific knowledge using data is absorbed into belief systems to discipline agents to become moral and honourable citizens [14,15]. I use ‘discourse’ to refer to the way personal and political value systems are communicated and reproduced in the wider social context [16] through social narratives; they enable and constrain ways of thinking, writing and speaking about a social object or practice within a particular historical limit [17]. The dominant discourse of early motherhood positions young mothers as irresponsible because they compromise the development agenda seeking to improve outcomes for adolescent girls and young women, especially in relation to health and education.

In South Africa, this discourse of ‘moral panic’ [18] was quintessentially expressed in 2015 by former-president, Jacob Zuma: ‘They [young teenage mothers] must be educated by government until they are empowered and they can take care of their kids, take them to Robben Island or any other island, sit there, study until they are qualified to come back and work to look after their kids’ [19]. Mr Zuma reinforced the role of the state to educate women, suggesting that young women who transgressed socially sanctioned, normative reproductive timing are punished by removing them to the infamous island where anti-apartheid political prisoners were held. He positions young women as powerless and without agency.

An emerging discourse in response to this dominant representation of early motherhood engages with more complex dynamics attending to social and cultural contexts, economic imperatives, and gender. This emergent discourse shifts the focus from the lens of socio-economic disadvantage associated with early motherhood [20–22], to explore the interplay of structural factors that impact on the lives of young women, and the decisions they make regarding an early, unintended pregnancy [21,23–29]. In listening to the voices of young women, it recognises their agency, and resists framing them as powerless. Studies illustrate how some young women see motherhood as aspirational in a context of limited opportunity [30], as a means of achieving adulthood [31], and of finding a purpose in life or having someone to love [12,32,33]. Accordingly, I use the term ‘young mother’ to avoid the pejorative framing associated with the term ‘teenage mother’ [31,34–36].

### Aim of study

This PhD study aimed to understand how young mothers exercise agency in relation to their own lives and the health and wellbeing of their babies. I explored the experiences of young women from pregnancy to motherhood, and focused on how institutions like health services, schools and family mediated their experiences. I aimed to understand the agency and aspirations of young mothers in a context where young women are often positioned as having limited or no agency when macro-structural forces like age, race, class, gender and culture operate to minimise their power and autonomy.

### Theoretical framework

I draw on the work of the sociologist Anthony Giddens’ [37] ‘theory of structuration’ to explore the dialectic between structure and agency. Structures are the spaces within which actors move, are spread across time and space, and exist beyond the scope of the individual agent [37,p.204]. Structures consist of ‘constraining and enabling’ [37,p.25] rules and resources that create the foundation of social institutions. Agency refers to the purposeful action of individuals. Agents – with hopes and aspirations – operate within the boundaries of the social structures which influence their objective social situation and their agency within this; power mediates the dialectic between structure and agency. Power refers to the ability to assert one’s will [38].

## Methods

This ethnographic study was situated in the township of Alexandra, Johannesburg, South Africa. The term ‘township’ is a relic of apartheid terminology and refers to underdeveloped urban settlements once reserved for Black, Coloured and Indian people who worked in or near the cities.

**Figure 1.**
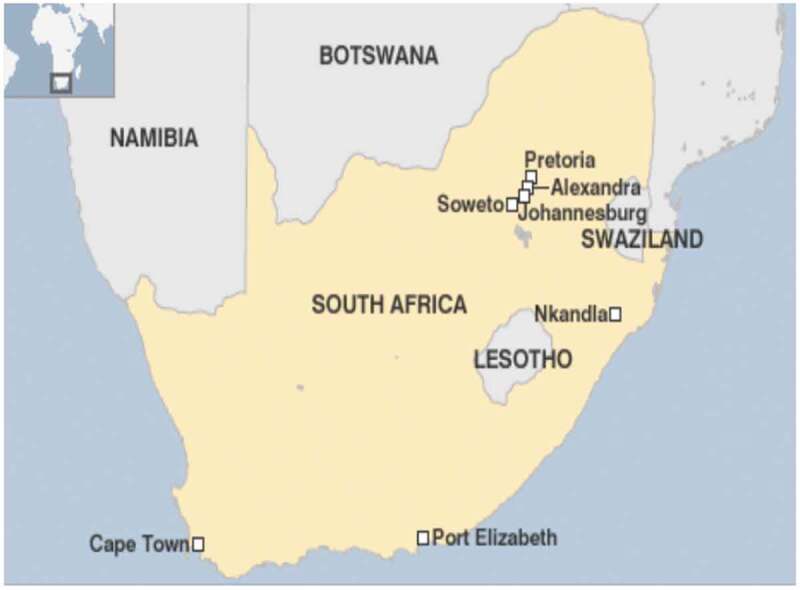
Map of Alexandra township in Johannesburg, South Africa [39]

**Figure 2.**
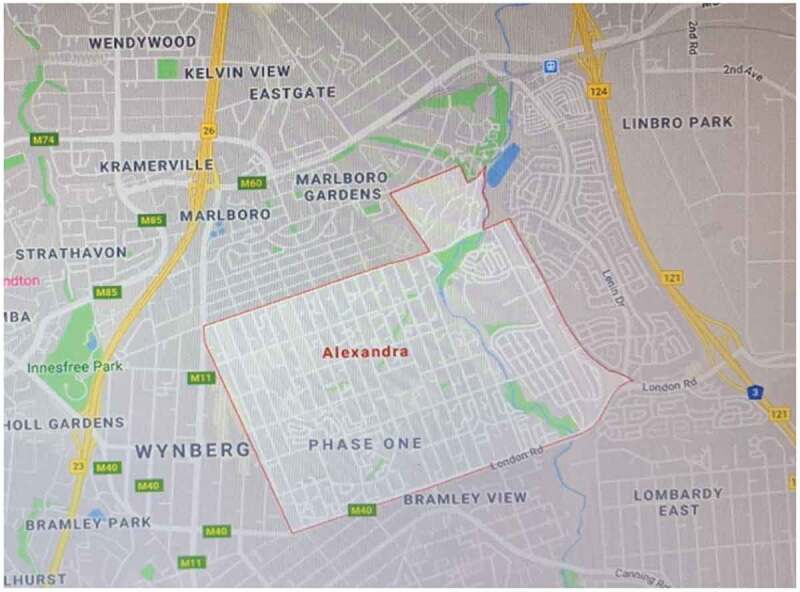
Map of Alexandra in the City of Johannesburg [40]

Many townships persisted after apartheid and remain underdeveloped, poorly serviced and largely constituted of poor, Black South Africans. Alexandra was established in 1912 and is one of the few townships situated in the middle of the urban hub, bordering one of the wealthiest suburbs (Sandton) in South Africa (Figure 1). Alexandra (Figure 2) is 6.91 square kilometres with a population of 179 624 residents [41] and is characterised by high population mobility, fluid households and sporadic unrest.

The study was qualitative and longitudinal. Between September 2016 and 2017, I used a health centre as an ethnographic base and recruited young mothers visiting the Expanded Programme for Immunisation (EPI). Sampling was designed to ensure maximum variability across age (ten women each aged 18, 19 and 20) and experience: in school, returning to school, out of school; with and without partners; living with and without families; in employment, looking for work, unemployed, choosing not to be employed [42,p.60–61]. The first level of sampling was purposive, supported by staff at the health facility, where I introduced the study to women and their babies, established interest and sought permission to interview young mothers between 18 and 20 years. I chose the latter range of teenage years because pregnancy numbers increase rapidly from age 17 to 20 in South Africa [7,8]. The second level was snowball sampling, where young mothers who were already participants, and other community members, helped with recruitment.

I recruited 30 young mothers; I interviewed all once (n = 30), nine twice (n = 9) and six three times (n = 6) over the course of one year. I requested repeat interviews using mobile telephone numbers provided by young mothers with their consent. I proceeded with follow up interviews with those who responded; I did not apply pressure to young mothers who did not respond. I also interviewed four significant others identified as the person who offered significant support to the young mother and her baby: two mothers, a grandmother, and the father of a baby. Significant others were associated only with young women who had been interviewed three times, illustrating that time and trust are needed to build relationships with research respondents. In two instances I was invited by young women to speak to their significant others at their homes. At the health centre, I interviewed six nurses who provided services to young women. In this paper, I present only data analysed from interviews with young mothers. Interviews were conducted in English; all participants refused the use of a translator to use the local language isiZulu. All interviews were recorded and transcribed verbatim. All young mothers were accompanied by their babies during the interview. Interviews with the different respondent groups were analysed in peer reviewed publications: three journal articles, a case study, and the thesis. Each publication dealt explicitly with the structural domains of families [43,44], health services [45] and schools [46].

My interactions with young mothers required reflection on my positionality and the power relations inherent in the research experience [47,p.10]. As an older, middle-class, Indian woman, I was visible as an outsider in the township, which possibly created curiosity (outsider) and safety (outsider, older) for young women. I drew on elements of our shared identity as a woman and mother by identifying and engaging with their experiences, as well as creating ease and comfort by playing with their babies during the interviews. Furthermore, young women expressed some comfort from speaking to someone about their experiences, which they had rarely or never had the opportunity to do.

Narrative enquiry was the primary methodological approach, paying attention to the narratives of young women and exploring their lived experience of negotiating early, unintended pregnancy. This approach privileges the actor in her context [48,p.326], allowing me to foreground the experiences of young women whose voices remain largely absent in representations of early motherhood. The interviews focused on three main areas aligned to the theoretical framework: pregnancy and birth (health services); education and income generation (school and post-school); and support networks (families). I used a semi-structured interview schedule for all first interviews; these provided a baseline for analysis, and allowed for comparison in the sample. Subsequent interviews drew on the first interview, and deepened the inquiry in relation to context and experience. I paid attention to *who* supported young mothers and *how* they offered support, and how structural institutions like health services and schools mediated experiences of early, unintended pregnancy and motherhood.

Data collection and analysis were simultaneous and iterative [42,p.65]. All transcriptions were verified by me listening to recordings and reading transcripts; this provided a first step in analysis and allowed for iterations in interview themes, additional probing and exploring emergent themes not considered at the study outset. For example, after the initial review of data, I paid greater attention to the composition and spatial distribution of households and families, which enabled me to explore the kinship networks of young mothers. I used qualitative data software MaxQDA™ for coding all interviews and began with open coding, then identifying a priori and emergent themes and sub-themes. I used structuration theory to analyse how agents (young mothers) negotiate their identities as young pregnant and parenting women within the structural spaces they occupy (families, health services, schools) to achieve their aspirations [42]. Excel™ was used to analyse basic demographic data, to keep track of the major themes, and for simple descriptive statistics. Table 1 below provides a brief summary of some of the characteristics from the sample of young mothers (n = 30).

**Table 1.** Summary of respondent characteristics

Respondent Characteristics	Variable	Number of Mothers
Number of children	One child	27
	Two children	2
	Three children	1
Relationship status	In a relationship with the father	18
	Not in a relationship with the father	11
	Did not disclose	1
Material support from father	Father provides material support	20
	Father does not provide material support	10
Termination of pregnancy	Did not consider termination	18
	Considered termination but did not access	12
Education status at pregnancy discovery	In school	24
	Completed school	6
School attendance during pregnancy	Continued school	13
	Temporarily drop out of school	6
	Permanently drop out of school	5
Child Support Grant (CSG)	Received the CSG	19
	Did not receive the CSG	9
	Not eligible for CSG	2

## Results and discussion

I present a synthesis of the study as it relates to the main research question of how young mothers exercise agency in relation to health services, education and caregiving, and how they mediated structural constraints to achieve their aspirations. I present the results with an integrated discussion that relates the findings to the literature, and begin each section with a concise overview of the relevant policy. Although presented as three discrete sub-headings, the results are inter-related across structural domains.

**Figure 3.**
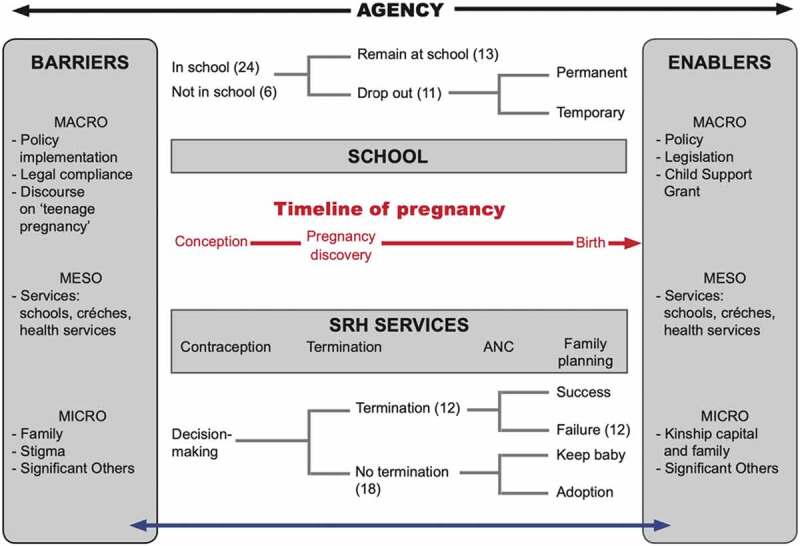
A structure-agency conceptual framework for early motherhood

The conceptual framework, Figure 3, provides an overview of the study and illustrates the intersection of agency and structure (theoretical framework) with the study aims. Figure 3 reflects the agency of young mothers explored along a timeline from conception to motherhood, highlighting key decision-making moments. I use the decision-making processes of young women as a conceptual tool to understand how agency is operationalised. Agency is represented as a continuum – subtle and dynamic in relation to time and place. The boxes to the left and right of the figure represent the structural barriers and enablers to the agency of young women. Figure 3 also provides a synthesis of key findings from the study.

In the section that follows, I use the conceptual framework (Figure 3) to show how young mothers navigate the world of early childbearing in their negotiations and mediations with the health system, schools, and families. I use Giddens’ [37] structuration theory to describe the duality between the agent (young mother) and her actions (agency) within the structural spaces she occupies at particular moments in time, showing that agency is exercised and/or constrained along a continuum from conception to motherhood. This provided a framework to illustrate how policy, legislation, services, and families act as barriers and facilitators to mediate the agency of young women. By drawing attention to the social and structural constraints that impact on how young women engage with their circumstances after an unplanned pregnancy, structuration theory provides a useful conceptual tool to further understand the sexual and reproductive lives of young women.

Individual narratives in the results highlight important themes in the data; quotes are attributed by pseudonym and age at first interview, unless otherwise indicated.

### Navigating the health system

South Africa’s health policy for young women is framed by HIV prevalence data. Young women are disproportionately affected with HIV prevalence levels of 5.6% in the 15–19 age group (males is 0.7%) and 17.4% for 20–24 aged women (men is 5.1%) [4]. Health policy targets the sexual and reproductive health and rights (SRHR) of adolescents, and focuses on access to youth friendly services [49,50], contraception [51] and provision of safe termination of pregnancy [52]. SRHR and Maternal and Child Health policy emphasises the importance of early ante-natal booking and access to emergency services particularly for young women under 16. Breastfeeding is a national health priority [53].

From pregnancy discovery to motherhood and after, young mothers navigate the health system at regular intervals: to confirm pregnancy; seek termination; access ante-natal, obstetric and post-natal care including family planning; and for immunisation thereafter. These visits are aligned to national policy directives. Young women who seek health services signal the success of policy to increase access to, and usage of, health services especially during pregnancy. An article [45] emanating from this study explores the experiences of young mothers and health service providers respectively receiving and providing health services and articulates how narratives of care and conflict are expressed in this experience. Below, I expand on key themes associated with the experience of young women at health services, focusing on termination, ante-natal care, family planning and breastfeeding.

#### Termination of pregnancy

In this sample, 12 young women (Table 1) had considered terminating their pregnancies. Some young women were dissuaded from termination by family, friends and partners because of fear and ethical dilemmas associated with termination. For example, Fikile (18) was pressurised by her boyfriend to keep her baby:

He said I must keep the baby but I kept on saying I don’t want this baby, I’m gonna [terminate]. He said don’t do that please, this is my first and last child, I don’t want to lose this thing. I say no, I’m going to do it. What will people say? He was like no, don’t listen to other people.”

Fikile succumbed and listened to her partner.

Young women also described that termination services were not available at health services at which they presented, fear of health care provider responses, or receiving inaccurate information from providers including being asked for consent from parents – which is illegal [52]. These young women were met with a health system with poor or no capacity to perform termination, or with health care providers who failed in their responsibility to advise and refer appropriately. Young women’s rights in relation to reproductive choice were thereby denied, and their agency in relation to termination was constrained by wider structural barriers. The ineffective delivery of termination services despite South Africa’s legislative framework is well documented [54–58], and these findings are consistent with this. They reflect the importance of improving service delivery as a mechanism to reduce rates of early, unintended and unwanted pregnancies in South Africa.

#### Ante-natal care

At the study health centre, young women did not report poor treatment or age-related discrimination. However, neither they nor health care providers offer evidence of any targeted support during pregnancy or after, despite their age and associated health-related vulnerabilities. Examples of abuse and discrimination were provided by some young mothers who began ante-natal care outside Alexandra; Sesi, aged 16 when attending her first ante-natal consultation in another province, was treated with hostility by nurses:

They [nurses] were always making things hard for me. How could you get pregnant so young? Now you have HIV and you are still a baby. How are you going to grow and your dreams are … you don’t have dreams anymore now. There is no more future for you. [42,p.2]

This experience was not unique, and there is a vast literature on the failure of South Africa’s public health service to provide youth sensitive services [45,59–62]. Sesi (18), resentful of the attitude of the nurses, resisted their projections of her future: ‘There is no more future for me? Like really, are you kidding? A baby is not the end of the world. That’s what I realised after everything I went through for the past two years.’ Her ongoing attendance at ante-natal care and her use of the health service required negotiating a hostile environment, testimony to her agency as a young woman and her commitment to her own health and the health of her baby. After experiencing discrimination from school too, Sesi and her baby relocated to Alexandra to be with her mother, who supported her daughter manage some of the challenges of early motherhood.

#### Family planning

Avoiding repeat pregnancy is a public health goal and contraception after birth is therefore critical. Young mothers reported that at family planning services six weeks post-partum, they were offered the injectable contraceptive Implanon or the three-month injection (Depo Provera) with little or no inquiry into their individual family planning needs or preferences [45]. For example, some young women preferred to get a monthly period, and health care providers did not always take this seriously, resulting in poor adherence, as elaborated by Bontle (19):

Yeah, then I started to feel that when I am on the injection I don’t get my monthly period, so I decided to stop because that was scaring me a lot. But when they told me that it is nothing to be worried about, it is just that … ja … but still, I didn’t come back to get the injection.

In addition, some young women feared weight gain from contraception. Pumla (19) explained: ‘The thing about family planning ma’am is that they [have] side effects, you get fat, you lose your shape.’ Pumla tried repeatedly to address her contraceptive concerns: ‘I went there the first time when I get sick, then they lectured me, and then after two months I went back. I only went three times.’ This resulted in a second pregnancy when Pumla was 19. This in turn led to her partner and his family withdrawing their support; they believed the second pregnancy was due to infidelity. When we met, Pumla was a single mother to two children under age three. The danger of not providing woman-centred family planning services and subsequent effects on adherence can lead to further unintended pregnancy.

Health worker attempts to get young women to use the contraceptive injection is entangled in an HIV-focused health system, with the belief that the injection also prevents HIV, and provides some explanation of why nurses are persuasive in dispensing the injection even when women are reluctant. However, a recent study trial, Evidence for Contraceptive Options and HIV Outcomes (ECHO) found no link between the contraceptive injection and HIV [63]. This confirms the need to provide wider contraceptive choices to women, to prevent a one-size-fits all approach used for ‘for too many black and brown women who want choices, dislike side effects and deserve equity with high-quality contraceptive programmes in high-income countries’ [64,65].

#### Breast is best

Public health messages like ‘breast is best’ and ‘breast is free’ [66,p.69] were well absorbed by young mothers; they aspired to be ‘responsible’ mothers through breastfeeding, regular health visits and immunisation for their infant. The slogan ‘breastfeed for a healthy nation’ [62,p.56] illustrates how public health messages become political, and how breastfeeding becomes synonymous with being a good mother [53,67]. Young mothers were aware of this, and often felt pressure to breastfeed and demonstrate responsible motherhood because they lacked financial resources for other feeding options – even when this clashed with aspirations like paid employment [68] or completing school. For young mothers at school, breastfeeding was therefore an act of good motherhood *and* deviant adolescence, and required managing the dual demands of breastfeeding and school and actively discarding their shame. Furthermore, young mothers aspiring to continue their education and/or employment, needed to engage in forms of co-operative caregiving, relying on the help and support from family, or from community caregiving structures like crèches. This eroded their agency in relation to feeding decisions, and having to make impossible choices between being a good mother and continuing education or employment [42,p.163–164].

### Reconciling education and motherhood

The South African Schools Act 1996 [69] guarantees the right of all South Africans to education. Given that approximately 28% of women under 20 have begun childbearing [8,p.12], it is critical that the education system creates an enabling educational environment for pregnant and parenting learners (scholar). The Draft National Policy on the Prevention and Management of Learner Pregnancy in Schools [70] guarantees pregnant and parent learners access to school and provides guidance on responding to learner childbearing. In this study, education was a dominant theme, as 24 young women were still in school when they became pregnant; five of these young women permanently dropped out of school at this point. Of the young women who continued school (n = 19), seven were between 15 and 17 years, and fifteen were between 18 and 20 years. The data from this sample reflects South African data: pregnancy increases substantially after age 17, with school completion often occurring after 18 [71,72]. For the 19 women who sought to reconcile education with pregnancy and motherhood; 13 remained in school during the pregnancy, while six temporarily withdrew from school (Table 1). An article from the study [46] expands on these pathways to school completion for young mothers. However, two points from the data deserve attention: one, young mothers experienced formal and informal forms of exclusion and discrimination at schools; and two, support from kin and others was critical in enabling school continuation.

#### Formal and informal discrimination at schools

Ridicule and shame experienced at schools, as others have elaborated [73–76] resulted in school discontinuation, as described by Lindi (20): ‘in my school they don’t want people who are pregnant … they would not allow me to write exams because I was pregnant.’ Bontle (19) reflected:

I would like to go back to school but oh, I think going back to school now is going to be very difficult for me. Because how do I cope with the other kids when I know that they too young, and I’m the oldest in the class and now I have a baby? So it will feel uncomfortable for me so I don’t really think I will go back to school.

Notwithstanding difficult experiences, young women often persevered with school motivated by fear of disappointing their families for whom education is aspirational:

No, the thing is at home my sisters don’t have matric, and my parents are unemployed, so I am the only person that had a chance to be in matric. And they are all looking up on me … I want to pass my matric so that maybe I will get a bursary and go to school so that I can support my family. So I don’t have a choice, I have to study very hard because they are all depending on me. I am the only that had a chance to go to matric. (Bertha, 18)

#### Support to continue school

Support from kin, and acts of kindness by teachers, were instrumental in keeping young mothers in school. Support from the school environment was a strong motivator in school continuation and in overcoming the multiple barriers they faced physically and emotionally.

They [teachers] were always supporting me, telling me when you are pregnant, don’t feel so ashamed of yourself, ya its part of life, you will get through it and those give me some stuffs like apples to make me smile because I was always so shy to walk, feeling like all the learners are watching me. (Tebogo, 19)

The kinds of support described by young women was often meagre, inconsistent, or not particular to pregnant and parent learners, but was nevertheless described as important in keeping them at school. Structural support like the Child Support Grant and early childhood centres gave young women some autonomy over their decisions around school completion and care for their babies [46].

Although policy and legislation were important in addressing various barriers to education, I identified that reconciling education with motherhood was difficult and required support and tenacity [46,58,77,78]. Emotional and physical strength were important to combine concomitant roles of learner and caregiver. Nolu (19) wrote her matriculation examination three weeks after giving birth:

I normally went to school at the time when, when we were going to write. Not early in the morning, um and like when we were writing at two, at two o’clock, I was going at one so that I can feed the baby.

Nolu’s story illustrates the difficulties of reconciling education with motherhood, particularly when breastfeeding is being established post-partum and decisions around care are being negotiated. Although Nolu completed her matriculation examination, she did not achieve the necessary grades for entry into a tertiary institution. In the study, only two of the 11 young mothers who completed matriculation after having a baby passed with grades required for tertiary education.

These findings on continuing or completing school suggest that shame and support work together to inhibit and encourage agency. They further illustrate how inescapable the negative discourse of early motherhood is, even in the South African context where education and motherhood is not uncommon. In reflecting on the impact of policy on keeping young pregnant and parenting women at school, the data show how education remains aspirational because national policy and subsequent discourse positions education as the silver bullet to eradicate poverty. Thus perseverance around completing school is higher than in other similar contexts, signalling some level of policy success in keeping girls in school. However, young women and their families aspirations around education and gaining a matriculation certificate (high school), reflects a wider systemic disconnect with South African reality, where further education and training levels are low and youth unemployment high – 33.2% of 10.3 million young people (15–24) are not in employment, education or training [79].

### Considering care

Considerations of care involved young mothers making decisions on how their babies would be cared for materially, physically and emotionally, while negotiating their aspirations for education and/or employment. Three sources of relational and structural support were important: the Child Support Grant, kinship capital, and support offered by the genitor.

#### The child support grant

The Child Support Grant is a means-tested monthly cash transfer of ZAR 440 (approximately USD 28) for children 0 to 18 years [80] and offers social protection to mothers and their children. This is particularly important for young women still at school or unemployed. Material support was a cause of great anxiety for all young mothers; the Child Support Grant was important in alleviating this anxiety for 19 young mothers, providing them with greater autonomy and ensuring food security. This was often supplemented with income support from their kin or the father of the baby.

The Child Support Grant gave young mothers greater agency over their caregiving decisions enabling access to paid care through crèches and community child caregivers when kinship care was not available. The availability of alternate care arrangements was important in this urban township because many households were women-headed with women responsible for income generation, thus making kinship caregiving less available. Furthemore, although there were créches in the community, young mothers often chose alternatives like paid caregivers or left school or work to care for their babies themselves, demonstrating agency in relation to the quality of care they desired. Betty (20), removed her son from the local créche because it was overcrowded and provided poor quality of care:

Yes, and when I entered the creche I will find the [my] child crying … I tell myself that [if] I tell the creche ‘I found the child crying’ they will drop mine [ignore her child]. The other child will come, they will take the one that is coming, and mine will cry. That is what I was thinking in that place. So I just stopped. Even if he can walk now, I won’t take him to that créche again.

She paid a community caregiver to care for her son while she worked, and was supported by her siblings to transport him. She demonstrated agency around the care she wanted for her son, but also limitations to her agency when confronting structural conditions like overcrowding and limited capacity.

#### Kinship capital

Kinship support, as provided to Betty and her son by her siblings, was highly significant for young mothers. Kinship refers to the ‘edifice of socially significant biological, genealogical and conjugal ties which every society constructs to order daily life, in the patterning of economic interdependence and patterns of co-residence’ [81,p.8]. In an article on kinship networks I explore this further; I use the term ‘kinship capital’ to refer to the multiple resources present within families, harnessed at different moments of disruption and allowing for resilience when circumstances change [44]. In this study, kinship capital varied at different times in response to the needs of young mothers for material, emotional, informational and caregiving support [44]. This was important when reconciling early motherhood, education and income generation.

Caregiving provided within kinship networks was strongly gendered, with older women shouldering the bulk of caregiving, emotional and material support provided to young women and their babies [43,82–84]. Women (mothers, grandmothers, aunts, sisters) were the most significant source of support for young mothers. Six young women identified the father of the baby as significant others. Consistent with other studies, mother-daughter dyads were the most common significant relationships [43]. Mothers were always disappointed to hear their daughters were pregnant, in spite of historically high levels of early childbearing and the culturally recognised value of motherhood. Charmaine (19) recalled her mother’s reaction:

I did not tell her myself; I asked my sister to tell her for me. Ja, ja, so she was so angry. She took something like maybe a month without talking to me without calling me, without taking my calls but she, she ended up calming down and then she spoke to me … I was feeling bad and I was feeling abandoned sort of, but I understood that it was my fault. Just that when she was she was not talking to me, I did not know when it was going to end you see, so I was stressed. Right, left and right because somehow I was doing matric and then I had to cope with the stress too.

In almost all instances mothers overcame their disappointment and provided care to their daughters and grandchildren. However, even when protected by kinship capital, young women internalised their families’ initial disappointment and adopted a punitive stance towards themselves. As found in other contexts, they believed that taking the bulk of responsibility for the unintended pregnancy, or making amends by completing school or finding employment, was a way of learning a lesson for transgressing childbearing expectations [76,77,85,86].

#### Support from fathers

Twenty fathers (and their kin) (Table 1) were involved in varying degrees in providing material support and caregiving for their babies, even when they were no longer involved in relationships with the mother, thereby challenging dominant narratives of absent fathers [82,87,88]. Formal material support was often operationalised through the cultural practice of *inhlawulo*; payment of ‘damages,’ an amount paid by the genitor to the maternal family when a baby is conceived ex-nuptially, representing a commitment to the mother and baby. For fathers, economic constraints often prevented them from paying *inhlawulo*, thereby limiting their access to their children and restricting them from enacting new forms of masculinity that encourage healthy family relationships.

However, although *inhlawulo* was restrictive for fathers, it offered an opportunity of maternal gatekeeping to young mothers and so increased their agency; they and their kin decided if they wanted to ‘report the pregnancy.’

We had some conflict with his parents, ja, because like they wanted us to go to Soweto, this thing, this traditional thing of reporting you’re pregnant, when a girl is pregnant. They wanted us to go to Soweto and my grandmother was like no, because this baby was made here in Alex, he [genitor] has a family, a [paternal] grandmother here so we are going and report to the [paternal] grandmother, we are not going to Soweto. So we had a conflict about that, that is where we ended up breaking up because I was like no, you and your family, you have to understand I am staying with my grandmother, my grandmother cannot be travelling, coming to Soweto to report that I’m pregnant, no. (Ayanda, 19)

In this example of maternal gatekeeping, Ayanda, an orphan in the care of her grandmother, demonstrates agency through enacting fidelity to her grandmother. Her sense of responsibility to her grandmother means that Ayanda does not challenge her decision, even though this may not have been what Ayanda truly desired, illustrating how *inhlawulo* facilitated and constrained agency for young mothers and fathers.

## Conclusion

This study adds to an expanding scholarship of early motherhood. In exploring the intersection of agency and structure for young mothers in an urban area in South Africa, I highlight how young women ‘navigate,’ ‘reconcile,’ and ‘consider’ their circumstances during pregnancy and after, to reflect their agency and engagement [42,p.151] with their structural context. I highlight three themes. One, young mothers negotiate decision-making around pregnancy and parenting vis-a-vis three structures in which they are embedded. These are: the health service, as guardian and gatekeeper of the health and wellbeing for young mothers and their children; the education system, as a structural facilitator of future opportunities for young mothers; and family and kinship networks, as a locus of support. Policy and context determine how these structures work and how actors mediate their functioning. Two, young women exercise agency and navigate these structural contexts in varying degrees along a continuum influenced by power inherent and manifest in these structures. For example, kinship networks are dynamic, fragile, and malleable to changing conditions and circumstances. Education and health services are less fluid, are formally regulated by state policy and legislation; they enable interventions and changes but are also informally regulated by dominant discourses of early motherhood. My data show how the agency of young mothers is constrained by the power invested in structural institutions like health services and schools, confirming how their actions (agency) are mediated by structures [37]. Three, young mothers have multiple aspirations in relation to themselves and their babies (health and wellbeing, education, income, care) which require constant adjustment through pregnancy and after.

Aspiration and agency are contingent, and the narratives speak of a post-apartheid generation of young women aspiring for autonomy in a context shaped by economic and social forces that are empowering and disempowering [42,p.2]. It highlights that even when young women are constitutionally and legally protected, when pregnant they experienced discrimination, for example, being denied access to services like pregnancy termination and school attendance. However, the data also reflect the success of policy: young pregnant and parenting women adhere to policy guidelines for maternal and child health, and attempt in complex circumstances to continue education even when they experience shame and discrimination. The data further reflect the complexity in the relationships between service providers and users, often expressed and experienced as conflict and care [45], simultaneously entrenching and challenging the dominant discourse.

Structural enablers like the Child Support Grant and créches offer young women in this urban context opportunities for agency and provide them with caregiving options, and greater autonomy and economic independence in mediating the roles genitors play. Support provided by family, particularly women, play a critical role in empowering young mothers mediate structural and social challenges experienced in early, unintended motherhood. Kinship capital, a theoretical concept arising from this study, is a form of support that young mothers draw on at various decision-making moments. There is no single story of how early motherhood is experienced [89,90]. Young women practice ‘malleable motherhood’ [91,p.205] and dynamic social and cultural systems enable and constrain their agency as they navigate early motherhood.

## Recommendations

South African policy must target specific barriers to services experienced by young pregnant and parenting women. These include: expanding pregnancy testing outside health services for early pregnancy detection; increasing health system capacity to provide safe termination services; improving youth friendly, sensitive family planning services; extending social assistance *during* pregnancy. Stigma and discrimination by service providers must be eliminated – sensitising them to the challenges associated with early motherhood and developing their psychosocial skills to support young pregnant and parenting women would help eliminate one of the biggest structural barriers to accessing services.

## Study limitations

Simple statistical analyses presented from this qualitative study should be interpreted with caution, and were only used for comparison and reflection within this sample. Securing repeat interviews with young mothers in this urban area was challenging for multiple reasons including flux in living arrangements and barriers associated with mobile phone usage. A further limitation was the small number of significant others interviewed and reasons include: migration, employment, diverse household compositions, practicalities of setting up interviews, and reluctance by young mothers to refer because they feared violation of their privacy.

## Paper context

In South Africa, rates of early childbearing rise rapidly between 18-20 years. Studies have explored agency and decision-making with regard to contraceptive use, access to health services, and school completion, yet there is little research capturing the voices of young mothers, their decision-making processes and how they make meaning of their lives during early motherhood. This PhD study sought to address this gap, focusing on an urban context that has received less attention from South African scholars.
